# Sensitivity of El Niño intensity and timing to preceding subsurface heat magnitude

**DOI:** 10.1038/srep36344

**Published:** 2016-11-03

**Authors:** Joan Ballester, Desislava Petrova, Simona Bordoni, Ben Cash, Markel García-Díez, Xavier Rodó

**Affiliations:** 1 Institut Català de Ciències del Clima (IC3), Barcelona, Catalonia, Spain; 2 California Institute of Technology (Caltech), Pasadena, California, United States; 3 George Mason University, Fairfax, Virginia, United States; 4 ICREA, Barcelona, Catalonia, Spain

## Abstract

Despite extensive ongoing efforts on improving the long-term prediction of El Niño-Southern Oscillation, the predictability in state-of-the-art operational schemes remains limited by factors such as the spring barrier and the influence of atmospheric winds. Recent research suggests that the 2014/15 El Niño (EN) event was stalled as a result of an unusually strong basin-wide easterly wind burst in June, which led to the discharge of a large fraction of the subsurface ocean heat. Here we use observational records and numerical experiments to explore the sensitivity of EN to the magnitude of the heat buildup occurring in the ocean subsurface 21 months in advance. Our simulations suggest that a large increase in heat content during this phase can lead to basin-wide uniform warm conditions in the equatorial Pacific the winter before the occurrence of a very strong EN event. In our model configuration, the system compensates any initial decrease in heat content and naturally evolves towards a new recharge, resulting in a delay of up to one year in the occurrence of an EN event. Both scenarios substantiate the non-linear dependency between the intensity of the subsurface heat buildup and the magnitude and timing of subsequent EN episodes.

El Niño-Southern Oscillation (ENSO) is the dominant mode of interannual variability in the tropical Pacific^1^,^2^,^3^,^4^,^5^, and a major source of climate predictability, large-scale teleconnections, and impacts worldwide^6^,^7^,^8^,^9^. After several decades of intensive research, the main mechanisms explaining the dynamics of the phenomenon and the onset of El Niño (EN) and La Niña (LN) events are nowadays thought to be relatively well understood^3^,^10^,^11^,^12^. Since the eighties, when the first successful prediction of EN was issued^13^, steady improvements in the forecast of the phenomenon have led to a plateau at moderate skill at short and medium lead times^14^. Forecasts issued in spring or before are still generally unable to foresee whether an EN or a LN event will occur at the end of the year, the so-called spring barrier^15^,^16^. Although recent advances suggest that it is indeed possible to overcome this predictability limit, either with intermediate^6^, purely dynamical^7^ or purely statistical^17^ models, the value of a real-time operational scheme derived from these results remains to be fully accomplished.

Mounting evidence has favored the view of ENSO as a slightly damped periodic oscillation modulated by stochastic noise^12^,^14^,^18^,^19^,^20^, with its long-range potential predictability arising from the dynamics of the ocean subsurface^3^,^21^. The recharge oscillator^10^,^11^ is currently the leading paradigm for the link between the Tropical Heat Content (THC) in the ocean subsurface and ENSO variability. This theory is based on the delay between anomalies in the longitudinally-averaged depth of the thermocline, and therefore the THC of the basin, and eastern Pacific Sea Surface Temperatures (SST). This paradigm, which has been thoroughly validated in the literature^22^,^23^, describes a recharge (discharge) phase of Warm Water Volume (WWV) along the equator that leads EN (LN) events by about 2 to 3 seasons^21^,^24^. This stage is in turn preceded by the tilting mode^2^,^25^,^26^, which is characterized by an anomalous zonal gradient of the depth of the thermocline and is significantly associated with ENSO at long-lead times (e.g. the correlation between ENSO and WWV anomalies to the west of 155W is around 0.57 at lag 15 months, see ref. 3).

This description of the oscillation emphasizes the importance of the THC at different lead times for the growth and magnitude of subsequent EN events. Reference 27 found that the duration of a complete EN cycle is determined by the time required for the slow accumulation of warm water in the western Pacific. Reference 28 showed that the onset of EN usually begins during the second half of the year before the event, being identified as a fundamental process independent of the flavor of the episodes. A buildup in heat content along the equator has indeed preceded all the major EN events since 1980, and the magnitude of EN usually scales in proportion to the magnitude of the heat content buildup 2 to 3 seasons in advance^3^,^29^. Reference 30 used ocean-atmosphere coupled simulations to show that the prescription of a warm heat content anomaly immediately before the spring barrier can lead to the generation of a moderate EN event. These same simulations also showed that prescribed westerly wind bursts alone do not lead to significant EN anomalies, but they can instead greatly amplify heat content anomalies and generate a strong EN event when they are superimposed to an initially recharged ocean state^30^. Nonetheless, results also suggest that the heat content buildup may be a necessary but not sufficient condition for EN to occur^3^. For example, ref. 31 have recently shown that the 2014/15 EN event was stalled as a result of an unusually strong basin-wide easterly wind burst in June 2014, which discharged the basin, suppressed the Bjerknes feedback and impeded the growth of the strong episode that was expected for the end of the year. At that time, basin-wide uniform warm conditions were instead observed in the equatorial Pacific, which were followed by the record-breaking EN episode in boreal winter 2015/16.

To illustrate the importance of the THC at different lead times, Fig. 1a depicts the relationship between the December anomaly of the Niño3.4 (N34) Index and leading WWV anomalies^32^ in March of the same and/or the preceding year (here referred to as years 0 and −1, respectively) for all the EN episodes since 1980 (see ref. 33 for the classification of events). WWV is defined as the volume of water masses above the 20 °C isotherm within 120E-80W and 5S-5N. This figure confirms the widely accepted and well understood 9-month lead association between high WWV anomalies and EN events (blue circles), but importantly, it also shows that the same relationship holds for WWV anomalies one year earlier (i.e. 21 months before EN, green squares). Both relationships are strong, with correlations of 0.75 and 0.63 respectively, which increase to 0.85 (r^2^ = 72%) when March WWV anomalies are averaged for both years (red diamonds). Note that this double relationship is in general also valid for all years since 1980 (Fig. 1b), with only one major exception: WWV was largely positive 21 months before the 1998 LN event, given that it was preceded by the very strong 1997 EN episode and its associated 9-month leading recharge phase in spring 1997 (dashed lines in Fig. 1b). In this way, when the 1998 LN event is not taken into account, the correlation between the December N34 Index and 21-month leading WWV anomalies is equal to 0.55 for all the years since 1980 (green squares in Fig. 1b).

Given the strong relationship observed between this very long-lead heat buildup and subsequent EN events here we use a state-of-the-art Earth System Model to explore the response to a decrease or increase in the magnitude of the heat content stored in the ocean subsurface (see Methods). We performed 11 sets of ensemble experiments, with initial conditions corresponding to an early phase of the onset of an EN episode in March of year −1 (i.e. lead time of 21 months). Previous studies have explored this relationship at shorter lead times of up to one year, through statistical analyses of observational data (e.g. ref. 3) or through numerical experiments (e.g. ref. 30). As we wanted to specifically study the dynamics of very strong EN events, we prescribed anomalous conditions to mimic as close as possible an episode of magnitude similar to the recent 2015/16 event (i.e. N34 = +2.8 °C). In each of these sets of ensemble experiments, the intensity of the subsurface warm anomaly was decreased (negative sign representing a discharge in heat content) or increased (positive sign or recharge) by ±20%, ±40%, ±60%, ±80% and ±100% (Supplementary Figure 1a,c,d,f) relative to the unmodified reference (REF) simulation ensemble (Supplementary Figure 1b,e). As such, differences among experiments are explained by both the magnitude of the initial subsurface heat content and the strong coupling between the ocean and the atmosphere that characterizes the dynamics of ENSO. We note that unlike in ref. 30 we only prescribe anomalies in the ocean subsurface, and therefore the atmosphere is only indirectly modified when the readjustment of the ocean affects the ocean surface and the interaction between the ocean and the atmosphere (see Supplementary Figures 2–4).

Figure 2 shows the longitude-time Hovmöller diagram of equatorial potential temperature and zonal current anomalies at the level of the thermocline for experiments representative of the different types of ocean responses to the prescribed anomalies, Fig. 3 shows the corresponding anomalies of equatorial SST and zonal wind stress, and Fig. 4 the anomalies of equatorial Sea Surface Height (SSH) and surface zonal currents. As additional information, the Supplementary Material includes the vertical profiles and horizontal maps of these variables for the time steps in which the recharge (Supplementary Figures 5–7), EN (Supplementary Figures 8–10), discharge (Supplementary Figures 11–13) and LN (Supplementary Figures 14–16) phases of the oscillation are observed in the REF ensemble.

The REF ensemble is found to correctly reproduce the main features of a canonical ENSO oscillation (Figs 2, 3, 4d). It is initially characterized by easterly wind and cold SST anomalies in the central Pacific and the generation of the subsurface heat buildup in the western Pacific, which peaks in spring of year −1 (i.e. beginning of the simulations). Reference 26 showed that meridional and eastward heat advection due to equatorward subsurface mass convergence and transport along the equatorial undercurrent contribute to this long-leading subsurface warming at 170E-150W, while surface horizontal convergence and downwelling motion have a leading role in subsurface warming in the warm pool. Westerly wind anomalies appear at the beginning of the following year, when the warm waters start to propagate to the eastern Pacific along the equatorial thermocline as downwelling Kelvin waves. Some few months later, in spring of year 0, the warm anomalies reach the eastern Pacific subsurface during the basin-wide recharge phase of the ENSO oscillation, which is immediately followed by the beginning of the warming of the ocean surface. Equatorial SST anomalies exceed the +1 °C threshold in the central and eastern equatorial Pacific between the summers of this and the following year (i.e. year +1). The eastward surface current anomalies rapidly become westward just after the peak, favoring the decaying phase of EN. The warm phase of ENSO is associated with the shoaling of the thermocline and the accumulation of subsurface cold waters in the western Pacific, which propagate to the east as upwelling Kelvin waves once the EN event starts to decay and the zonal wind anomalies become easterly in the western Pacific. A LN event develops as soon as the subsurface cold anomalies reach the eastern Pacific, although the magnitude of its peak is found to be approximately one half of the magnitude of the preceding EN episode.

When the initial heat buildup is modified by ±40%, the growth and propagation of the subsurface warm anomaly, as well as the subsequent onset of the EN event, remain very similar in timing and approximately proportional in magnitude to that shown in the REF ensemble (Figs 2, 3, 4c,e). This result is found to be valid both for surface and subsurface temperatures, as well as for surface winds and ocean currents, showing that the same dynamical mechanisms operate during the phases of the oscillation that precede and follow the peak of EN events. In the particular case of the +40% (−40%) ensemble, the magnitude of the event is nearly proportionally larger (weaker), with the anomaly of the N34 index reaching +3.5 °C (+2 °C) and representing an increase (decrease) of about 30% relative to the REF ensemble.

Further increasing the initial heat buildup up to 80% of the REF simulation induces some interesting differences (Figs 2, 3, 4f). The excess heat in the western Pacific is released and quickly starts to warm the eastern part of the equatorial Pacific subsurface. Nevertheless, the surface warming at the end of year −1 is found to be weak and uniformly distributed along the equatorial Pacific. This configuration does not favor the activation of the Bjerknes feedback and therefore the Walker circulation remains in a neutral phase (i.e. westerlies in the west but easterlies in the east), resulting in weak EN conditions (N34 < +1 °C). As a result, the accumulated heat is not discharged towards higher latitudes, and therefore this initial EN-like event only represents a step in the slow but steady warming of the basin, characterized by an initial warm base state of the equatorial Pacific, and enhanced by strong westerly wind anomalies progressing to the east throughout year 0. The subsequent EN event is found to be very strong (N34 = +4 °C) and followed by a strong LN event one year later.

The picture is however completely different in the ensemble of simulations in which the initial heat buildup is reduced by 80% (Figs 2, 3, 4b). The equatorial easterly wind anomalies (or, more precisely, the off-equatorial wind stress curl) observed before and after the beginning of the simulations are associated with the positive change rate in subsurface meridional convergence^10^,^11^. This tendency towards equatorward mass convergence is associated with upwelling of subsurface cold waters that favors the persistence of cold SST and easterly wind anomalies in the central Pacific, which in turn deepen the thermocline and accumulate subsurface warm waters in the western Pacific^26^. A weak LN event therefore develops at the end of year −1, which re-activates the generation of the subsurface heat buildup in the western Pacific. From this point onward, the evolution of the accumulated heat mimics that of the REF ensemble in terms of magnitude, timing, propagation and mechanisms, but with a one-year delay, leading to the growth of a strong EN episode that peaks in December of year +1 (N34 = +2 °C, i.e. same magnitude as in the −40% experiment, but one year later).

A similar evolution is found when the accumulated heat in the subsurface is completely suppressed in the −100% experiment (Figs 2, 3, 4a). In this case, however, given that the initial THC is weaker and the heat buildup is completely removed, the renewed recharge of the tropical Pacific results in a delayed EN event of smaller magnitude (N34 = +1 °C).

The relationship between initial THC and ENSO variability is summarized in Fig. 5. THC is defined here as the average heat content within 120E-80W, 5S-5N and the upper 300 m. Results show that the greater the initial THC, the greater its magnitude up to the spring of year 0, when the peak in THC is observed in the initially-recharged simulations (Fig. 5a). This dependency is strong during this initial period (correlation ≈1, pink line in Fig. 5b), but the recharge does not occur at the same pace (regression = 0.61 J/J in January of year 0, red line in Fig. 5b), as this process is faster in the initially-discharged simulations. The relationship between the initial and the time-varying THC becomes negative in autumn of year 0 (red and pink lines equal to zero in Fig. 5b), when all the simulations exhibit similar THC values (Fig. 5a). This includes the few initially-discharged experiments in which the heat content is still increasing at the end of year 0 (i.e. −100% and −80%), as well as all the other experiments (i.e. from −60% to +100%), in which the heat content is already being discharged at this point in time (Fig. 5a). The relationship then becomes negative, but it is still strong (correlation = −0.92 and regression = −1.35 J/J in June of year +1, Fig. 5b).

Results also show that the greater the initial THC, the greater the magnitude of the subsequent EN event (see Fig. 5c at the end of year 0). The relationship is again strong (correlation = 0.92 and regression = 1.56 °C/10^16^ J in December of year 0, blue and cyan lines in Fig. 5b), but not completely linear. On the one hand, in the initially-recharged simulations, negative feedbacks in the central and eastern Pacific limit the growth and magnitude of EN during its mature phase^10^,^11^,^34^,^35^,^36^, which explains why the N34 Index in December of year 0 is only +1 °C warmer in the +100% ensemble than in REF (Fig. 5c). On the other hand, the spread in the initially-discharged simulations is large, given that the Bjerknes feedback is not activated in some experiments (i.e. the N34 Index is equal to +2.8 °C in REF and negative in −100%, Fig. 5c). The relationship becomes negative in early summer of year +1 (blue and cyan lines equal to zero in Fig. 5b), when the transition between warm and cold conditions is found in most, albeit not all, simulations (Fig. 5c). Indeed, in the initially-discharged simulations, the timing of the peak of EN depends on the prescribed THC, with a clear phase-locking to the seasonal cycle that characterizes the jump between the winters of years 0 and +1^37^,^38^,^39^,^40^; Fig. 5d). Instead, all of the initially-recharged simulations are found to peak in October of year 0, defining a stepwise relationship between the initial THC and the timing of EN maxima (Fig. 5d).

The phase evolution of the system shows the traditional counterclockwise trajectory, in which the change rate of the N34 Index is approximately proportional to the THC, and the radius of the trajectories monotonically increases as a function of the initial THC (Fig. 5e, see also ref. 30 for a similar approach based on ocean energetics). Nonetheless, the trajectories of all the initially-discharged simulations tend to evolve towards the diagram values that correspond to the recharge phase of the REF ensemble (i.e. +0.5·10^16^ J ≤ THC ≤ +1·10^16^ J and −0.5 °C ≤ N34 ≤ +0.5 °C), and only then do they diverge to reach weaker N34 values than in REF. This indicates that, in our model configuration, the system compensates for the initially-prescribed reduction in heat content, and evolves towards a new recharge in THC and the generation of EN events through the memory of the system, regardless of the magnitude of the initial THC, and even when it is completely removed.

This general increasing trend of THC in the initial period of all the simulations is explained by the recharge theory, in which the off-equatorial wind stress curl is associated with the positive change rate in subsurface meridional convergence. In the initially-recharged simulations, this tendency towards the deepening of the thermocline in the central Pacific contributes to the transition towards the recharge phase that leads to EN by 2 to 3 seasons^10^,^11^. Instead, in the simulations in which the initial heat content has been completely or largely suppressed (i.e. −100% and −80%), the equatorward mass convergence is associated with the upwelling of subsurface cold waters that favors the persistence of cold SST and easterly wind anomalies in the central Pacific, which in turn deepen the thermocline and generate a new heat buildup in the western Pacific^26^,^27^. We must however note that, in a more general framework, the initial THC could increase at a different rate or even decrease if wind stress anomalies were also prescribed or a different time frame was chosen as initial conditions for the experiments.

The new recharge process in the −100% and −80% experiments can result in a delay in the occurrence of the EN event, which highlights the non-linear dependency between the intensity of the subsurface heat buildup and both the magnitude and timing of subsequent EN episodes. The numerical simulations reported here show that the accumulation of warm waters in the western Pacific determines the timing of the transition between LN and EN conditions, which is here seen to increase by one year when the initial subsurface heat is largely reduced. We found that the stepwise relationship between the initial THC and the timing of EN maxima also affects the magnitude of the events. For example, the EN episode at the end of year 0 in the −40% ensemble has similar magnitude to the event occurring one year later in the −80% experiment, because the longer timescale of the recharge process compensates the magnitude of the initial discharge. In this respect, our results provide new insight into the fundamental role of the ocean heat content, in this case at longer lead times than traditionally described, and therefore they have important implications for the understanding of the genesis of EN events, their dynamics and their predictability.

## Methods

Estimates of WWV for the 1980–2015 period were derived from the TAO Project Office of NOAA/PMEL, which are based on temperature analyses of the Bureau National Operations Centre at the Australian Bureau of Meteorology and profiles from TAO moorings, Argo floats and XBTs, with anomalies calculated after removing the 1980–2002 mean seasonal cycle^32^.

The model used in the present work, the Community Earth System Model (CESM) v1.2, is a global coupled model extending its predecessor, the Community Climate System Model (CCSM) v4, by incorporating new Earth system simulation capabilities^41^. The model configuration used here couples the latest version of the Community Atmosphere Model v5^42^ with the Parallel Ocean Program v2^43^, the Community Land Model v4^44^, the Community Ice Code v4^45^ and the River Transport Model^46^. The atmospheric model has a resolution of 2.5° in longitude and 1.875° in latitude, with 30 vertical levels. The ocean model uses a displaced pole grid with approximately 1° resolution in longitude and 0.5° in latitude, which is refined within the tropical band up to 0.25° at the equator. There are 60 vertical levels with the highest resolution (10 m) in the upper 150 m, and the lowest (250 m) in the deep ocean.

CESM was chosen to conduct the experiments reported here because CCSM was found to be one of the three best models in the simulation of the dynamic warm pool edge among 19 coupled ocean-atmosphere general circulation models from the Coupled Model Intercomparison Project Phase 5^47^. This feature corresponds to the easternmost edge of the western Pacific warm pool, the maximum in zonal salinity gradient and the area of surface ocean convergence, downward motion and advection and subsurface divergence^26^,^48^,^49^,^50^. The correct simulation of these dynamical features is seen to be key for the reproduction of the ENSO oscillation and the transition between events of opposite sign, including the generation of the heat buildup in the western Pacific and the eastward propagation of the accumulated heat^21^,^47^.

We performed 11 sets of ensemble experiments, with initial conditions corresponding to a very early phase of the onset of an EN episode, in March of the year preceding the winter peak of a strong warm event (i.e. a lead time of 21 months with regard to the December maximum), chosen from a reference 100 year spin-up simulation. This stage of the ENSO oscillation is characterized by cold LN-like conditions in the tropical Pacific and the generation of a subsurface heat buildup in the western tropical Pacific (Supplementary Figure 1b,e;^21^,^26^). In each of these sets of ensemble experiments, the intensity of the subsurface warm anomaly was decreased (negative sign representing a discharge in heat content) or increased (positive sign or recharge) by ±20%, ±40%, ±60%, ±80% and ±100%. The word “anomaly” in the experiments refers to the difference of a monthly value with regard to the long-term mean annual cycle computed from the REF simulation. We note that warm temperature anomalies were fully modified only in the inner three-dimensional box [120E-80W] × [10S-10N] × [50–200 m], and that this modification was linearly decreased to zero from the border of this box to the frontier of the outer box [100E-60W] × [15S-15N] × [20–300 m]. For example, Supplementary Figure 1a,d (Supplementary Figure 1c,f) show the initial condition in the −100% (+100%) experiment, in which the subsurface warm anomaly was suppressed (doubled). For the sake of clarity, we use the terminology ‘‘initially discharged’’ (‘‘initially recharged’’) simulations or ensembles to refer to the experiments with initially reduced (increased) subsurface warming, and we also compare the magnitude of these prescriptions by saying that the −100% (+100%) ensemble is ‘‘more initially discharged’’ (‘‘more initially recharged’’) than for example the −20% (+20%) experiment. Each set of experiments in turn consists of 10 simulations with slightly perturbed initial conditions, from which only the ensemble average is shown here.

## Additional Information

**How to cite this article**: Ballester, J. *et al.* Sensitivity of El Niño intensity and timing to preceding subsurface heat magnitude. *Sci. Rep.* **6**, 36344; doi: 10.1038/srep36344 (2016).

**Publisher’s note:** Springer Nature remains neutral with regard to jurisdictional claims in published maps and institutional affiliations.

## Supplementary Material

Supplementary Information

## Figures and Tables

**Figure 1:**
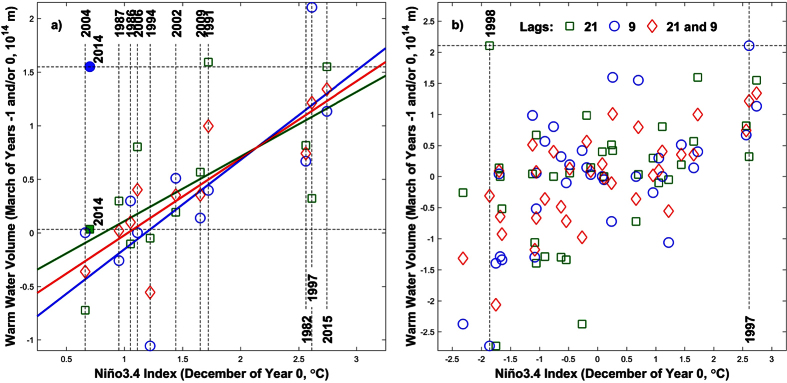
Observed relationship between the December anomaly of the Niño3.4 Index (in °C) and leading Warm Water Volume anomalies (10^14^ m) in March of the same year (blue circles), March of the preceding year (green squares), and the average of both years (red diamonds). Episodes in panel a correspond to all El Niño events since 1980 according to the NOAA’s Climate Prediction Center (vertical dashed lines, *33*), with colored straight lines depicting the linear fitting, while panel b shows values for all years in the 1980–2015 period. The 21-month (9-month) lag relationship corresponding to the neutral year of 2014 is additionally shown as a solid square (circle) in panel a. Warm Water Volume was computed as the volume of water masses above the 20 °C isotherm within 120E-80W and 5S-5N.

**Figure 2:**
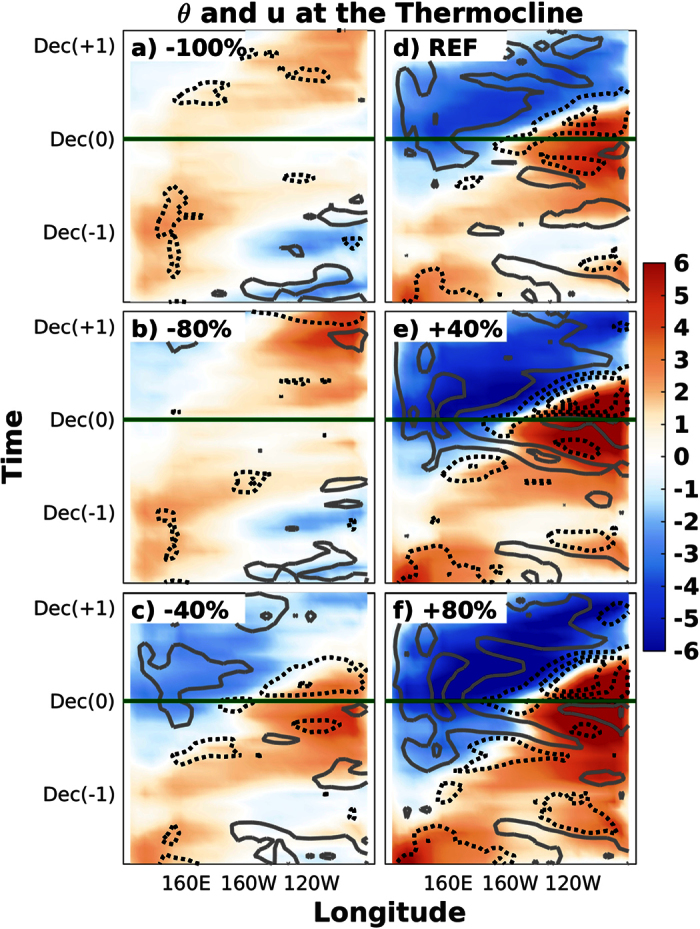
Longitude-time Hovmöller diagram of equatorial temperature (shading, in °C) and zonal current (contour, in m/s) anomalies at the level of the thermocline. Panels correspond to the −100% (**a**), −80% (**b**), −40% (**c**), REF (**d**), +40% (**e**) and +80% (**f**) experiments. The minimum contour is ±0.1 m/s and the contour interval is 0.2 m/s, with grey solid (black dashed) lines depicting positive (negative) anomalies. The horizontal green lines indicate the December month in which El Niño peaks in the reference simulation.

**Figure 3:**
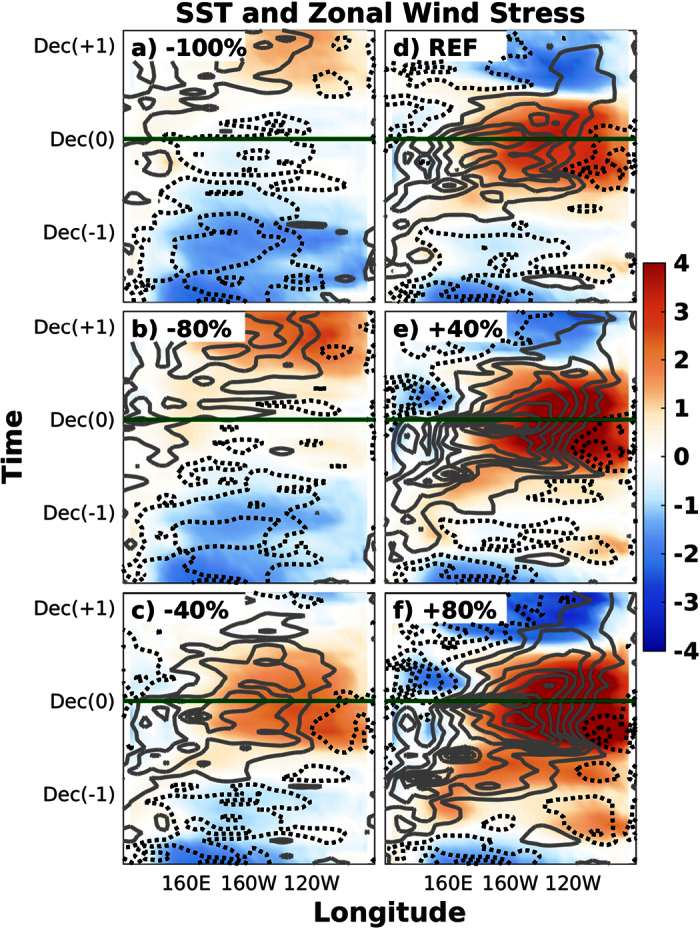
Longitude-time Hovmöller diagram of sea surface temperature (shading, in °C) and zonal wind stress (contour, in N/m^2^) anomalies. Panels correspond to the −100% (**a**), −80% (**b**), −40% (**c**), REF (**d**), +40% (**e**) and +80% (**f**) experiments. The minimum contour is ±0.005 N/m^**2**^ and the contour interval is 0.01 N/m^**2**^, with grey solid (black dashed) lines depicting positive (negative) anomalies. The horizontal green lines indicate the December month in which El Niño peaks in the reference simulation.

**Figure 4:**
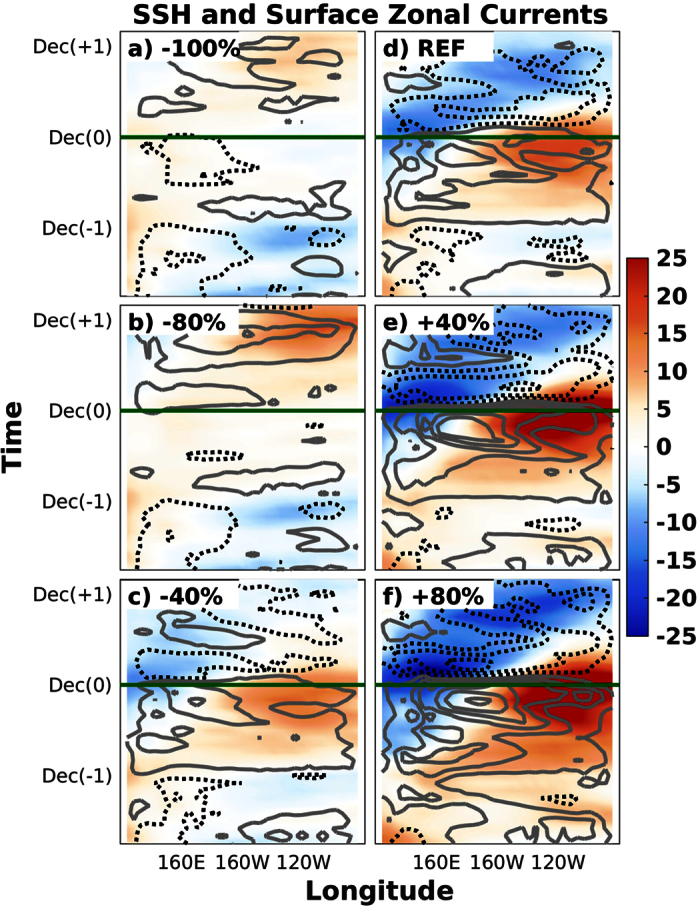
Longitude-time Hovmöller diagram of sea surface height (shading, in cm) and surface zonal current (contour, in m/s) anomalies. Panels correspond to the −100% (**a**), −80% (**b**), −40% (**c**), REF (**d**), +40% (**e**) and +80% (**f**) experiments. The minimum contour is ±0.1 m/s and the contour interval is 0.2 m/s, with grey solid (black dashed) lines depicting positive (negative) anomalies. The horizontal green lines indicate the December month in which El Niño peaks in the reference simulation.

**Figure 5:**
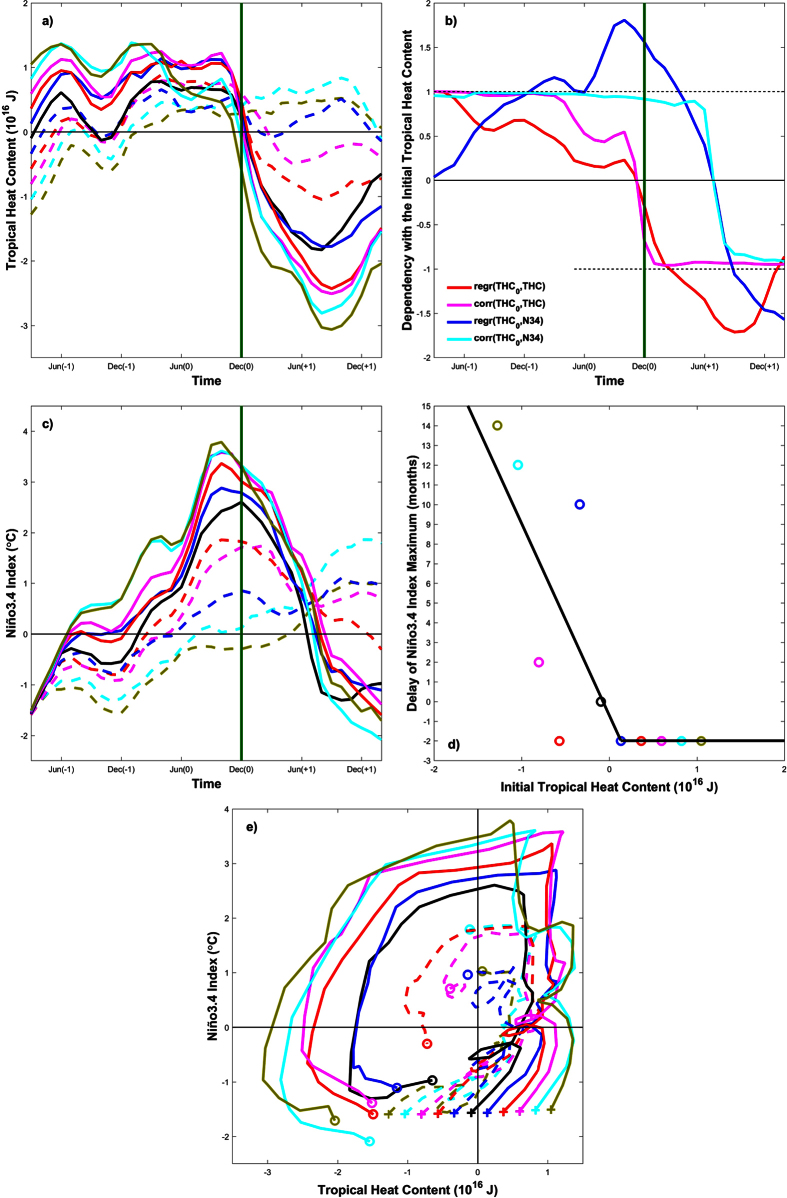
Relationship between Tropical Heat Content (10^16 ^J) and the Niño3.4 Index (°C). Panels (a,c,e) depict the evolution of Tropical Heat Content (THC) and the Niño3.4 (N34) Index. Crosses (circles) in panel (e) correspond to the initial (final) month of the model runs. Panel (d) shows the delay (in months) in the maximum of N34 relative to the December month in which El Niño peaks in the reference simulation (see vertical green lines in panels a–c). The black line in panel d shows the piecewise linear fitting between x- and y-axis variables. Curves and circles in panels (a,c–e) correspond to the −100% (dashed lime), −80% (dashed cyan), −60% (dashed magenta), −40% (dashed red), −20% (dashed purple), REF (solid black), +20% (solid purple), +40% (solid red), +60% (solid magenta), +80% (solid cyan) and +100% (solid lime) experiments. Panel (b) shows the regression coefficient (red and blue lines) and the Pearson correlation (pink and cyan) among model experiments between the Initial Tropical Heat Content (THC_0_) and THC (red and pink) and N34 (blue and cyan). Regression values in panel (b) are unitless for THC, and °C/10^16^ J for N34. THC was computed as the temperature average within 120E-80W, 5S-5N and the upper 300 m.
