# Supplementary material for: Sensitivity of El Niño intensity and timing to preceding subsurface heat magnitude
Source: Sci Rep. 2016 Nov 3;6:36344. doi: 10.1038/srep36344 (PMC5093742; doi:10.1038/srep36344)
Supplement: Supplementary Information [file srep36344-s1.pdf]

Title:

Sensitivity of El Niño intensity and timing to preceding subsurface heat magnitude

Authors:

Joan Ballester [1,2]
Desislava Petrova [1]
Simona Bordoni [2]
Ben Cash [3]
Markel García-Díez [1]
Xavier Rodó [1,4]

Affiliations:

[1] Institut Català de Ciències del Clima (IC3), Barcelona, Catalonia, Spain
[2] California Institute of Technology (Caltech), Pasadena, California, United States
[3] George Mason University, Fairfax, Virginia, United States
[4] Institució Catalana de Recerca i Estudis Avançats (ICREA), Barcelona, Catalonia, Spain

Corresponding author:

Joan Ballester
Institut Català de Ciències del Clima (IC3)
Carrer Doctor Trueta 203, 08005 Barcelona, Catalonia, Spain
Tel.: (+34) 935679977
Email: joanballester@ic3.cat

Submitted to Scientific Reports - Third Submission - Supplementary Material

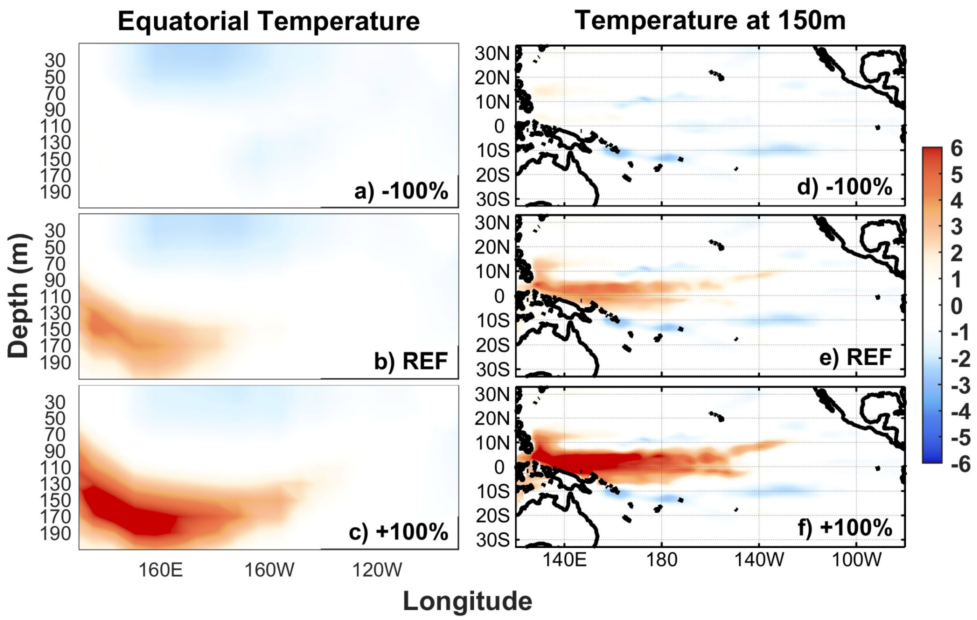

Supplementary Figure 1. Prescribed initial temperature (in °C) anomalies at the equatorial plane (a-c) and at 150 meter depth (d-f).

Anomalies correspond to the suppression of the heat buildup in the western Pacific subsurface (a,d; -100%), the reference run (b,e; REF) and the doubling of the heat buildup (c,f; +100%). Maps were generated with the mapping package M_Map v1.4h (www.eoas.ubc.ca/~rich/map.html).

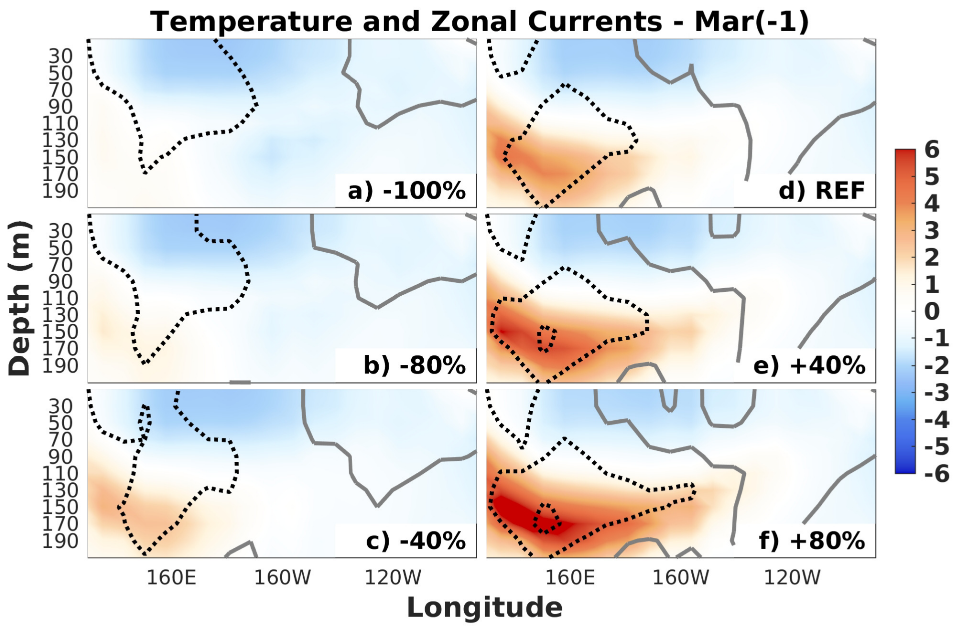

Supplementary Figure 2. Vertical profile of equatorial (2S-2N) temperature (shading, in °C) and zonal current (contour, in m/s) anomalies for the first month of the simulations (i.e. March of year -1). Panels correspond to the -100% (a), -80% (b), -40% (c), REF (d), +40% (e) and +80% (f) experiments. The minimum contour is ±0.1 m/s and the contour interval is 0.2 m/s, with grey solid (black dashed) lines depicting positive (negative) anomalies.

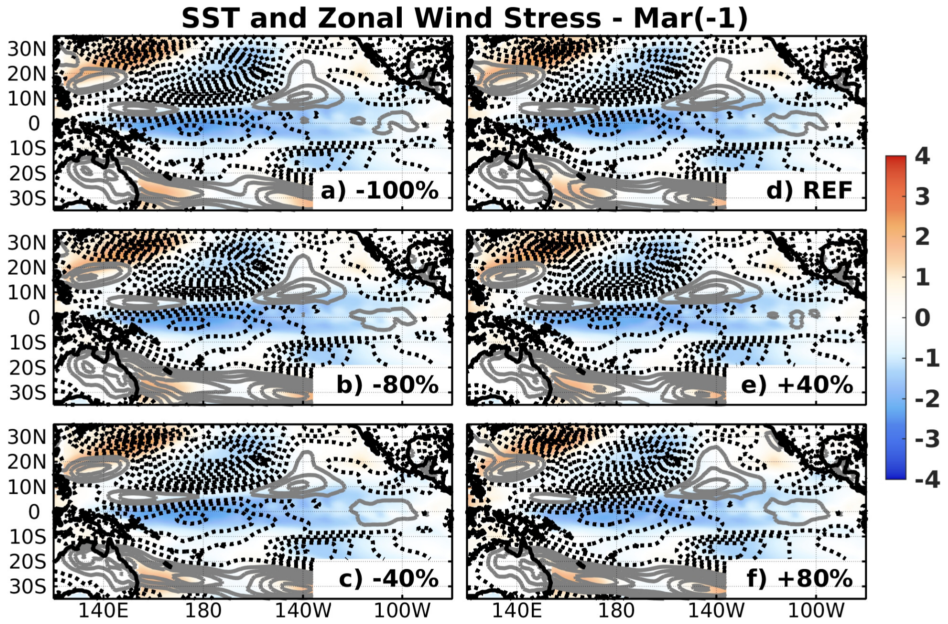

Supplementary Figure 3. Map of sea surface temperature (shading, in °C) and zonal wind stress (contour, in N/m²) anomalies for the first month of the simulations (i.e. March of year -1). Panels correspond to the -100% (a), -80% (b), -40% (c), REF (d), +40% (e) and +80% (f) experiments. The minimum contour is ±0.005 N/m² and the contour interval is 0.01 N/m², with grey solid (black dashed) lines depicting positive (negative) anomalies. Maps were generated with the mapping package M_Map v1.4h (www.eoas.ubc.ca/~rich/map.html).

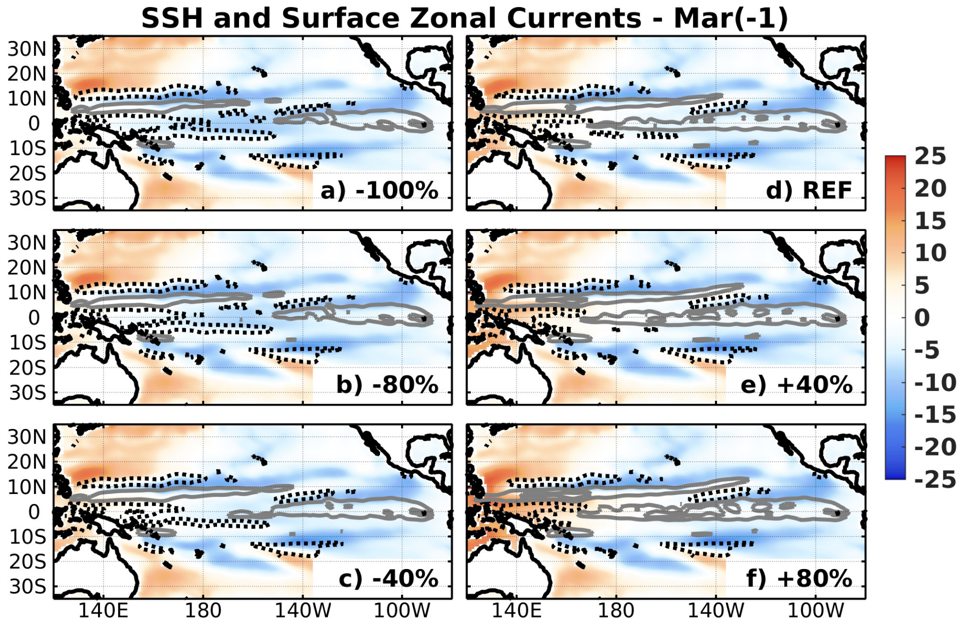

Supplementary Figure 4. Map of sea surface height (shading, in °C) and surface zonal current (contour, in m/s) anomalies for the first month of the simulations (i.e. March of year -1).

Panels correspond to the -100% (a), -80% (b), -40% (c), REF (d), +40% (e) and +80% (f) experiments. The minimum contour is ±0.1 m/s and the contour interval is 0.2 m/s, with grey solid (black dashed) lines depicting positive (negative) anomalies. Maps were generated with the mapping package M_Map v1.4h (www.eoas.ubc.ca/~rich/map.html).

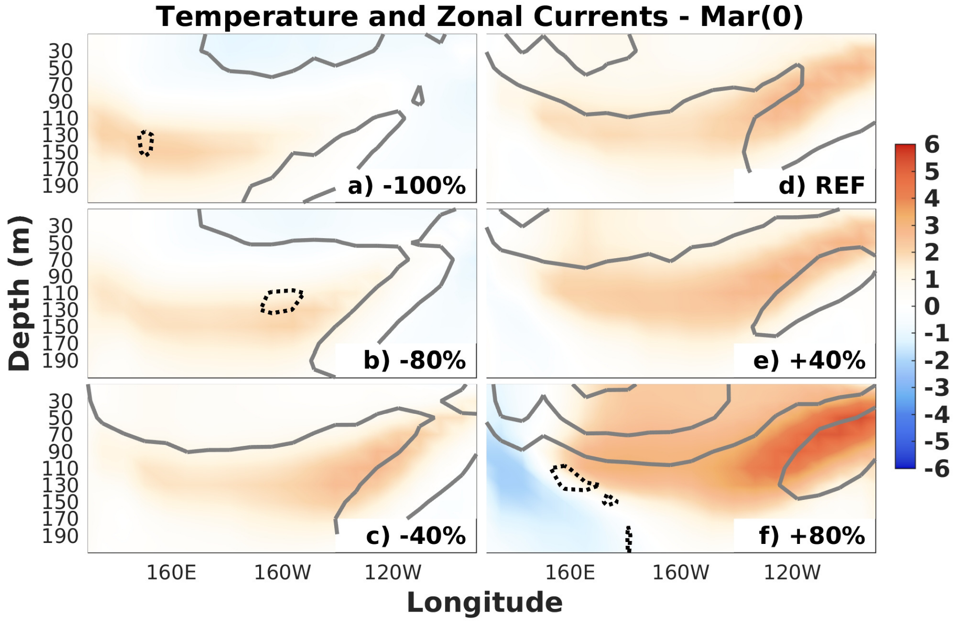

Supplementary Figure 5. Same as Supplementary Figure 2, but for the March month preceding the peak of El Niño in the reference simulation (i.e. March of year 0).

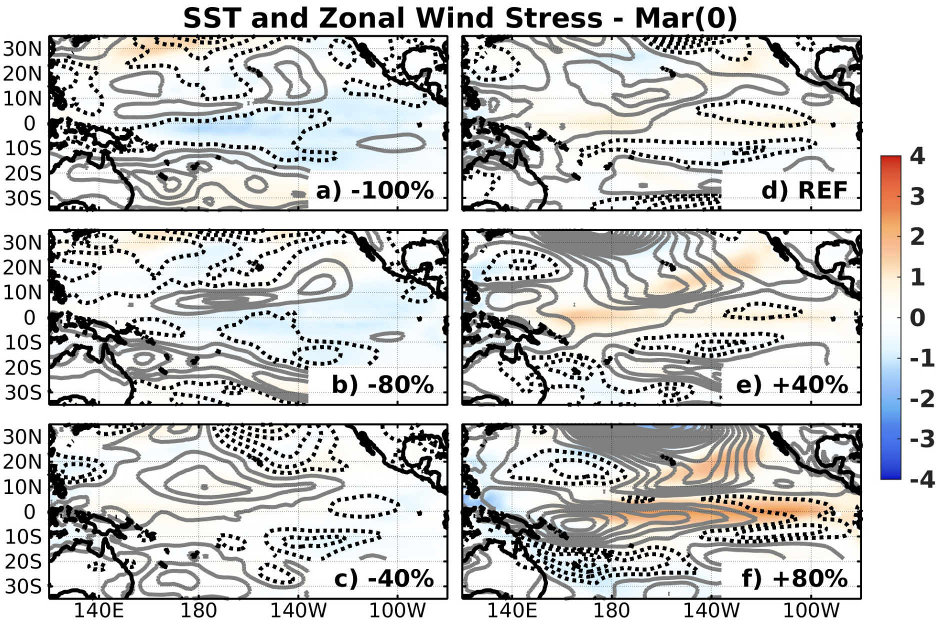

Supplementary Figure 6. Same as Supplementary Figure 3, but for the March month preceding the peak of El Niño in the reference simulation (i.e. March of year 0).

Maps were generated with the mapping package M_Map v1.4h (www.eoas.ubc.ca/~rich/map.html).

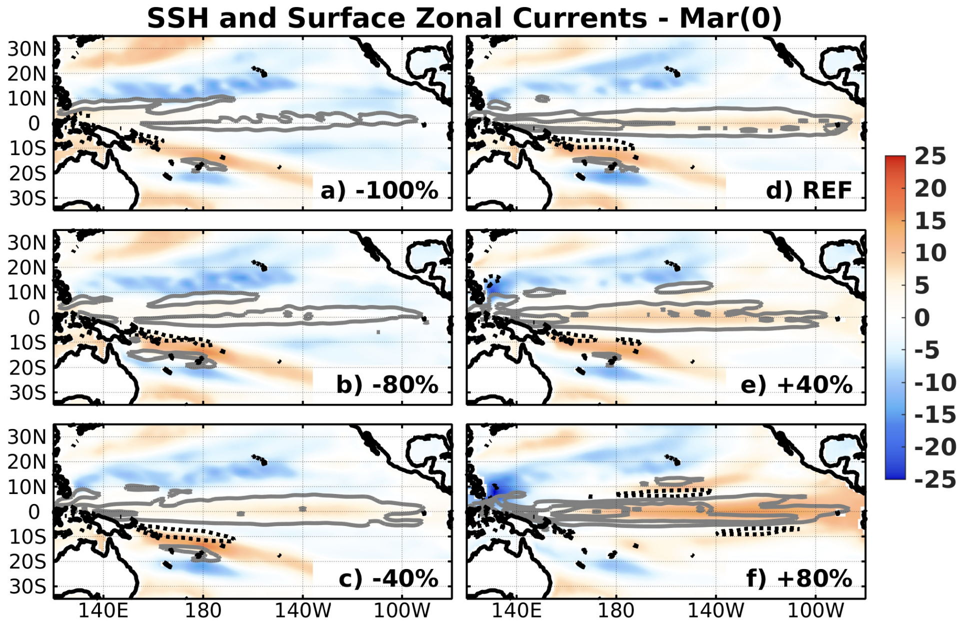

Supplementary Figure 7. Same as Supplementary Figure 4, but for the March month preceding the peak of El Niño in the reference simulation (i.e. March of year 0).

Maps were generated with the mapping package M_Map v1.4h (www.eoas.ubc.ca/~rich/map.html).

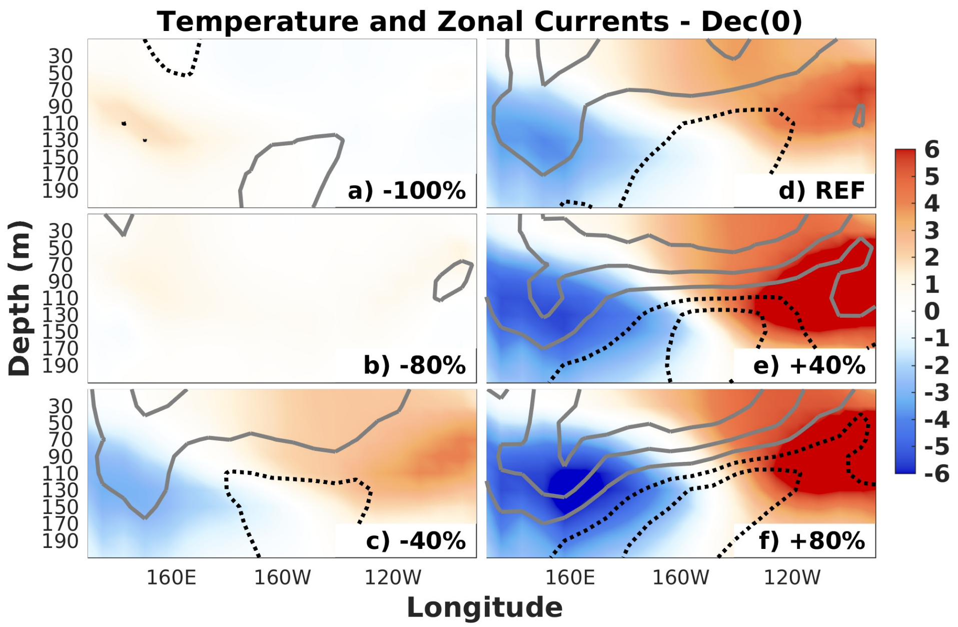

Supplementary Figure 8. Same as Supplementary Figure 2, but for the December month in which El Niño peaks in the reference simulation (i.e. December of year 0).

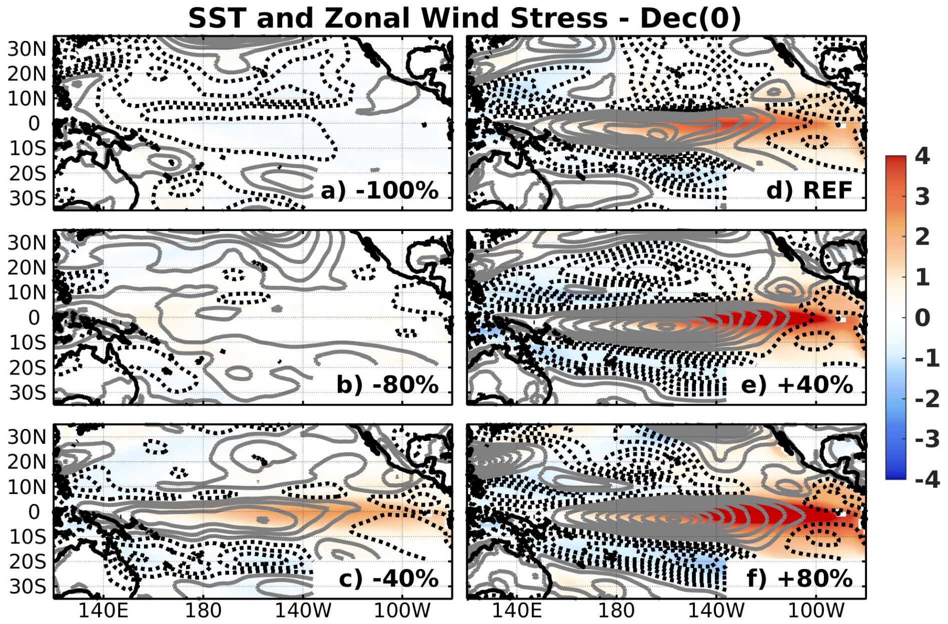

Supplementary Figure 9. Same as Supplementary Figure 3, but for the December month in which El Niño peaks in the reference simulation (i.e. December of year 0). Maps were generated with the mapping package M_Map v1.4h (www.eoas.ubc.ca/~rich/map.html).

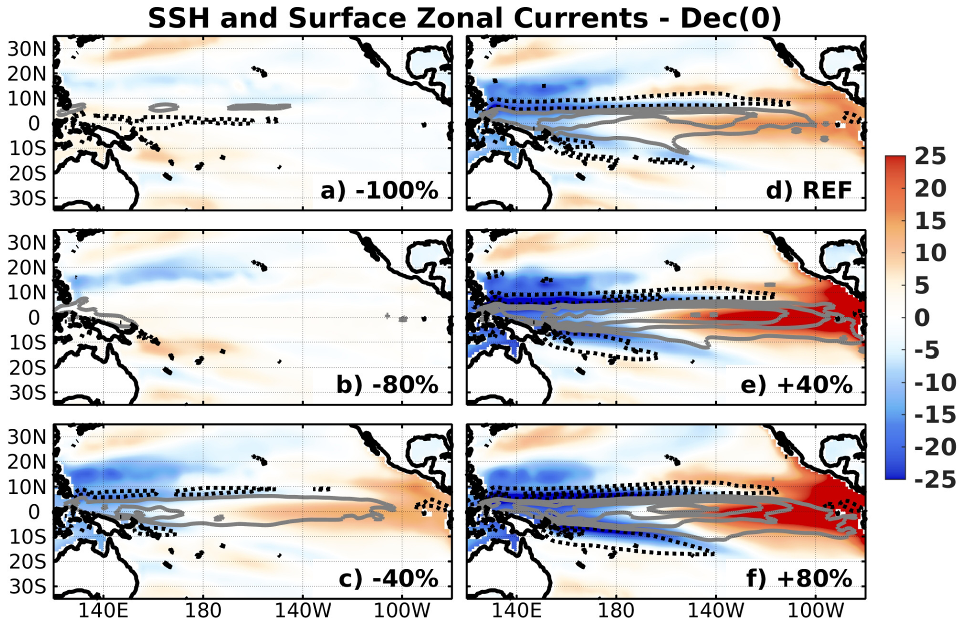

Supplementary Figure 10. Same as Supplementary Figure 4, but for the December month in which El Niño peaks in the reference simulation (i.e. December of year 0).

Maps were generated with the mapping package M_Map v1.4h (www.eoas.ubc.ca/~rich/map.html).

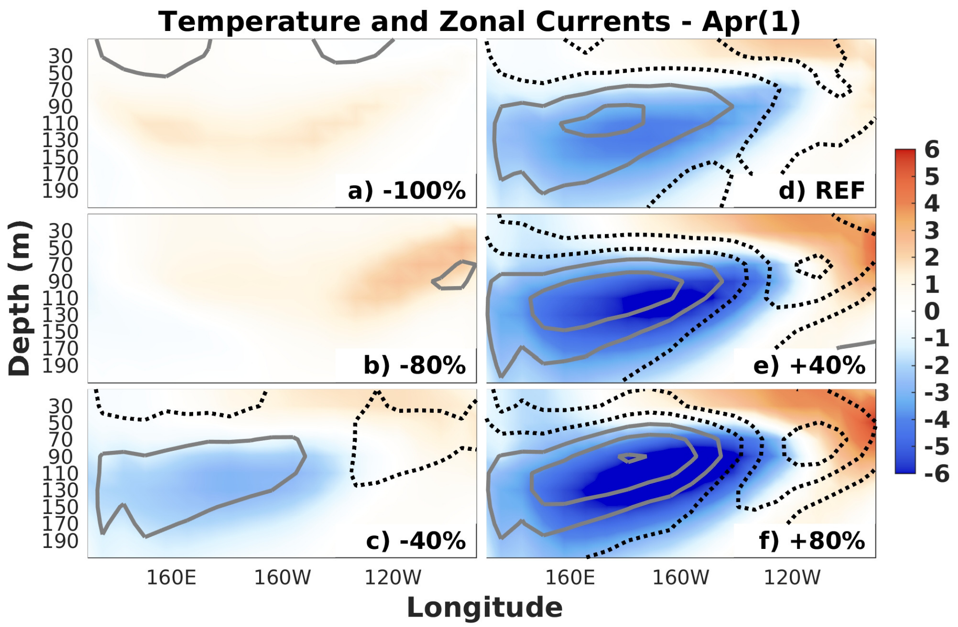

Supplementary Figure 11. Same as Supplementary Figure 2, but for the April month after the peak of El Niño in the reference simulation (i.e. April of year +1).

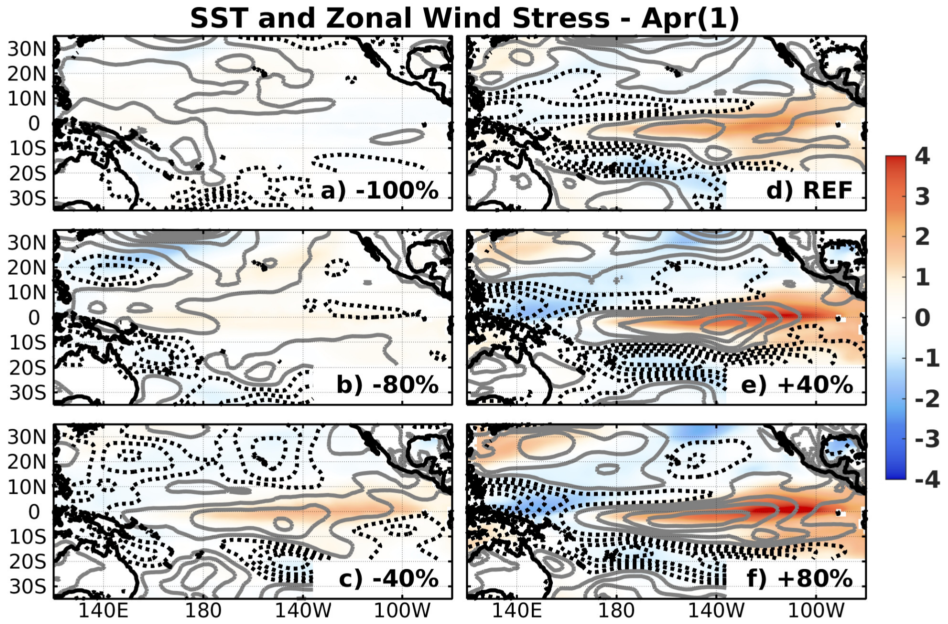

Supplementary Figure 12. Same as Supplementary Figure 3, but for the April month after the peak of El Niño in the reference simulation (i.e. April of year +1).

Maps were generated with the mapping package M_Map v1.4h (www.eoas.ubc.ca/~rich/map.html).

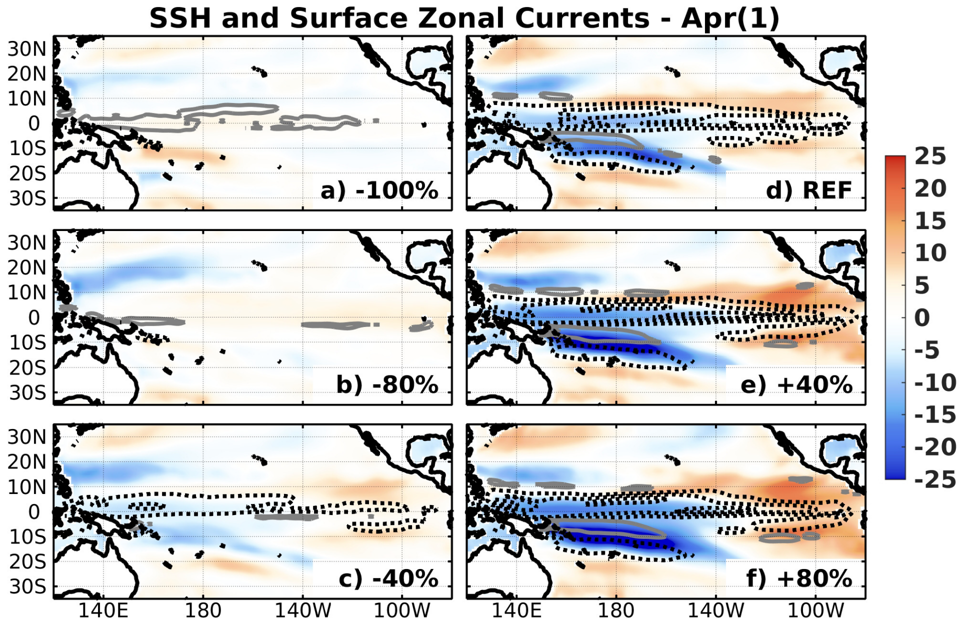

Supplementary Figure 13. Same as Supplementary Figure 4, but for the April month after the peak of El Niño in the reference simulation (i.e. April of year +1).

Maps were generated with the mapping package M_Map v1.4h (www.eoas.ubc.ca/~rich/map.html).

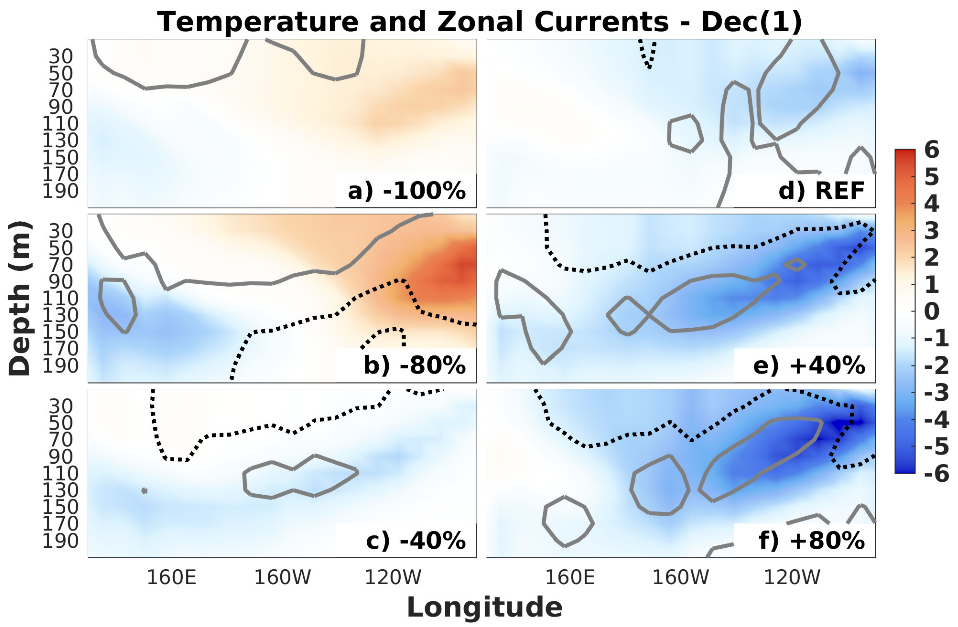

Supplementary Figure 14. Same as Supplementary Figure 2, but for the December month after the peak of El Niño in the reference simulation (i.e. December of year +1).

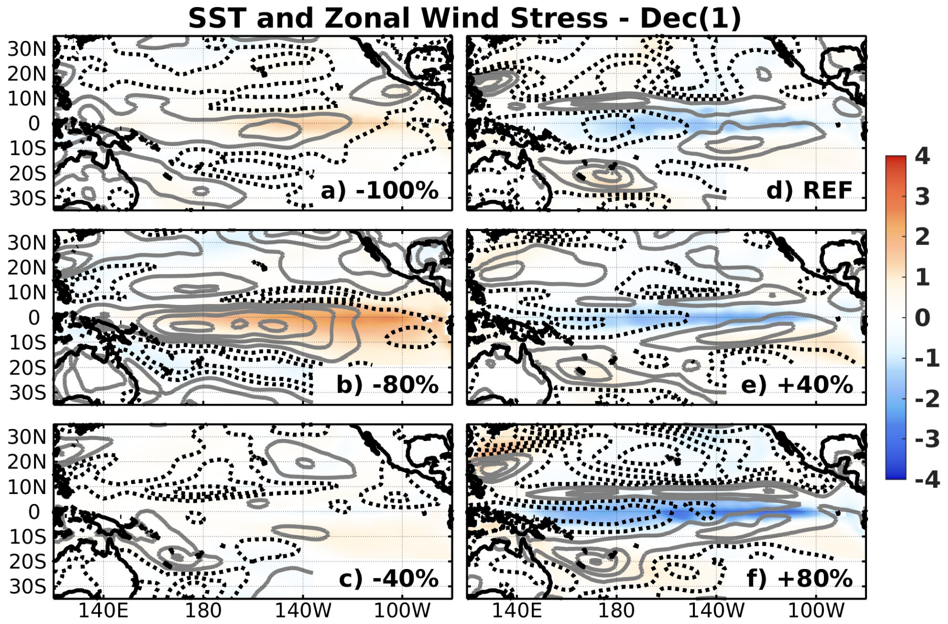

Supplementary Figure 15. Same as Supplementary Figure 3, but for the December month after the peak of El Niño in the reference simulation (i.e. December of year +1).

Maps were generated with the mapping package M_Map v1.4h (www.eoas.ubc.ca/~rich/map.html).

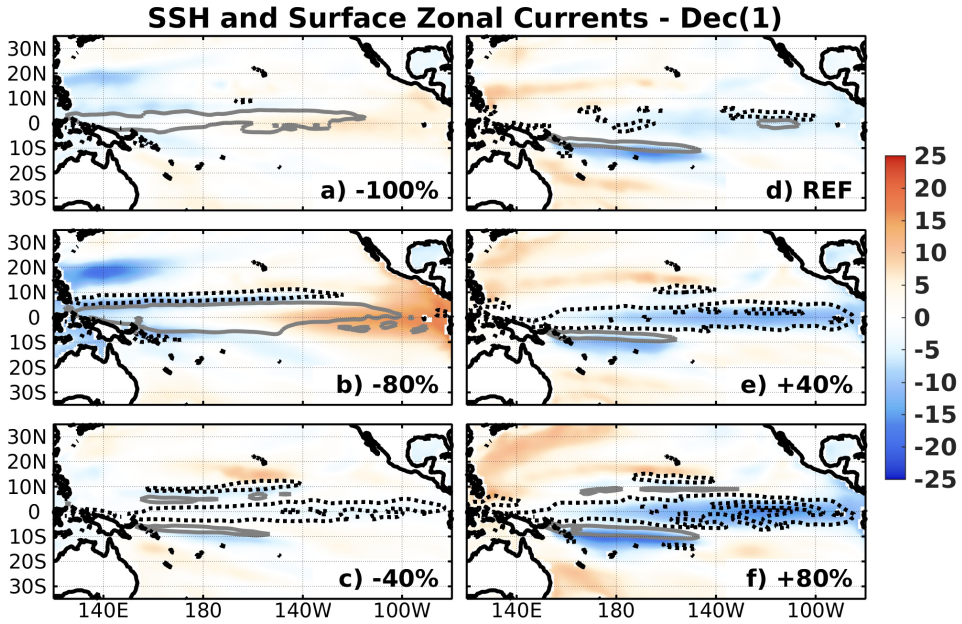

Supplementary Figure 16. Same as Supplementary Figure 4, but for the December month after the peak of El Niño in the reference simulation (i.e. December of year +1).

Maps were generated with the mapping package M_Map v1.4h (www.eoas.ubc.ca/~rich/map.html).
